# Reclaiming the Microbial Battlefield: Adjuvant Strategies to Overcome Antibiotic Resistance

**DOI:** 10.3390/microorganisms14030609

**Published:** 2026-03-09

**Authors:** Jing Sun, Ding Li, Tong Wu, Zengqi Yang, Dongyang Ye, Kangkang Guo

**Affiliations:** 1 College of Veterinary Medicine, Northwest A&F University, Yangling 712100, China; sunjing_jy@163.com (J.S.); yzq1106@nwsuaf.edu.cn (Z.Y.); 2 Experimental Animal Center, Northwest A&F University, Yangling 712100, China; 3 Key Laboratory of Animal-Derived Bacterial Resistance Monitoring (Co-Construction), Ministry of Agriculture and Rural Affairs, Yangling 712100, China; 4 Key Laboratory of Ruminant Disease Prevention and Control (West), Ministry of Agriculture and Rural Affairs, Yangling 712100, China

**Keywords:** antibiotic adjuvants, antimicrobial resistance, drug synergy, metabolic reprogramming, high-throughput screening

## Abstract

The escalation of antibiotic resistance constitutes a significant global health crisis, urgently demanding innovative strategies to restore and amplify the effectiveness of our existing antimicrobial arsenal. This review systematically explores the diverse landscape of antibiotic adjuvants, beginning with an elucidation of their classifications and profound mechanisms of action. We meticulously detail various categories of these agents, encompassing both synthetic compounds and naturally derived molecules, highlighting their crucial functions in potentiating antibiotic activity through mechanisms such as enhancing bacterial cell permeability, modulating the expression of resistance-conferring genes and optimizing drug–target interactions. Furthermore, we critically assess current screening methodologies, particularly the advancements in high-throughput approaches, and their implications for the identification and validation of novel adjuvant candidates. The review underscores the potential of antibiotic adjuvants as a promising frontier in our global endeavor to overcome bacterial resistance and ensure the sustained utility of antimicrobial medicine.

## 1. Introduction

The advent of penicillin through Alexander Fleming’s serendipitous discovery in 1928, rapidly followed by its widespread clinical application, irrevocably revolutionized infectious disease management, solidifying antibiotics’ status as a cornerstone of modern medical intervention in human and veterinary therapeutics [1,2]. However, the profound and rapidly accelerating challenge of antibiotic resistance (AMR), intrinsically linked to the very mechanisms of antibiotic action and powerfully driven by bacterial evolutionary pressures, now threatens to render these life-saving drugs ineffective [3]. In response, global public health bodies are urgently advocating for novel antibiotic discovery to counter the escalating threat from a growing array of multidrug-resistant (MDR) pathogenic bacteria [4,5]. The grim reality of highly drug-resistant bacterial infections, for which treatment options are severely limited or entirely absent, is becoming increasingly commonplace. Indeed, the rise of critical pathogens such as vancomycin-resistant *Enterococci* (VRE), methicillin-resistant *Staphylococcus aureus* (MRSA), and carbapenem-resistant *Enterobacteriaceae* (CRE) has propelled AMR into a dire global crisis [6,7]. The emergence of novel resistance determinants, exemplified by the 2009 discovery of the metallo-β-lactamase gene *bla*_NDM-1_, has further intensified this crisis [8,9]. Alarming statistics reveal that AMR claims approximately 700,000 lives globally each year [10]. Unmitigated, current projections predict that by 2050, AMR could result in a cumulative 300 million premature deaths and incur a total global economic loss of up to $100 trillion. [11,12]. The relentless ascent of MDR, extensively drug-resistant (XDR), and pan-drug-resistant (PDR) “superbugs” presents formidable challenges to the clinical management of bacterial infections and poses a grave threat to global public health security [13].

While the pursuit of entirely novel antimicrobials remains a vital long-term strategy to circumvent resistant genes and eradicate refractory bacteria, it is severely hampered by substantial financial investments and protracted timelines, which can extend up to 15–20 years from initial discovery to regulatory approval [14,15,16,17]. Alarmingly, since the 1970s, the decelerated pace of new antibiotic development, compounded by the ceaseless emergence of novel resistance genes, has rendered the selection of effective antibiotic treatments progressively more arduous [1,18,19,20]. This disconcerting reality—where the evolution of resistance outpaces the introduction of new drugs—underscores the urgent need for innovative and economically sustainable strategies to combat bacterial resistance effectively [21,22,23].

Consequently, a critical approach to countering AMR involves developing innovative combination therapies that strategically integrate existing antibiotics with potent adjuvants, thereby effectively restoring and augmenting antimicrobial efficacy against resistant bacterial strains [15,24,25]. Antibiotic adjuvants, defined as compounds possessing minimal intrinsic antibacterial activity, offer a highly promising therapeutic avenue by either directly disrupting bacterial resistance mechanisms or significantly enhancing the performance of co-administered antibiotics [16,26,27]. Their development has rapidly gained prominence as a pivotal research strategy aimed at circumventing the widespread proliferation of drug-resistant pathogens. These molecular interventions provide a sophisticated and nuanced approach, specifically designed to bypass bacterial resistance by precisely targeting the molecular mechanisms that compromise antibiotic effectiveness [28,29,30]. This review provides an in-depth, contemporary analysis of antibiotic adjuvant classifications, comprehensively elucidating their diverse molecular mechanisms of action. Also, it critically evaluates systematic screening methodologies designed to identify promising therapeutic candidates. By synthesizing cutting-edge research findings and spotlighting innovative molecular strategies, this work aims to significantly advance the global scientific discourse on combating antimicrobial resistance and fostering a sustainable future for antibacterial therapy.

## 2. Categorization of Antibiotic Adjuvants

**Figure 1:**
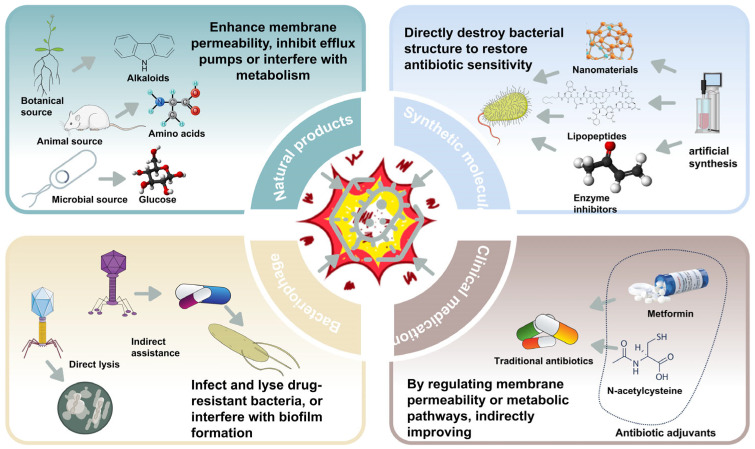
Main Classification and Mechanisms of Action of Antibiotic Adjuvants.

Antibiotic adjuvants, often conceptualized as “resistance circuit breakers” or “chemosensitizers”, represent a pivotal pharmacological strategy designed to precisely modulate antimicrobial efficacy through multifaceted molecular mechanisms [15]. Unlike conventional antimicrobial agents, which directly target microbial viability, these compounds do not inherently eliminate bacterial populations. Instead, they orchestrate intricate cellular perturbations that dramatically restore antibiotic susceptibility in otherwise resistant bacterial strains [17]. While traditional adjuvants often possess minimal intrinsic antibacterial activity and do not inherently eliminate bacterial populations on their own, the broader combinatorial strategy includes agents like bacteriophages and specific antimicrobial peptides. These agents may possess intrinsic lytic or membrane-disrupting activity, but they function synergistically alongside conventional antibiotics to actively dismantle resistance mechanisms and restore microbial susceptibility. The intrinsic value of antibiotic adjuvants lies in their capacity to systematically dismantle bacterial defense architectures through a multi-pronged attack. This includes, but is not limited to, biofilm disruption, enhancement of membrane permeability, metabolic recalibration, and synergistic modulation of pharmacodynamic interactions [31]. Such sophisticated interventional strategies fundamentally transcend traditional antimicrobial paradigms, offering a nuanced and powerful approach to circumventing emergent antimicrobial resistance. By re-conceptualizing antibiotic deployment as a multidimensional molecular dialogue with the pathogen, adjuvants provide an indispensable framework for therapeutic optimization. Reflecting this complexity, antibiotic adjuvants encompass a wide spectrum of molecular types, each endowed with distinct mechanisms and properties that underpin their efficacy in confronting antimicrobial resistance (Table 1, Figure 1).

### 2.1. Natural Products: Unleashing Nature’s Adjuvant Potential

Natural products, long a cornerstone of pharmacopoeia, represent an exceptionally rich and diverse reservoir for novel antibiotic adjuvants. These compounds, derived from botanical, animal, and microbial sources, offer a sustainable and often mechanistically distinct approach to combating antimicrobial resistance (AMR).

#### 2.1.1. Phytochemicals Derived from Botanical Sources

Botanical sources are a prolific and highly diverse wellspring of bioactive compounds with profound therapeutic potential. Phytochemicals, abundant in nature, have garnered significant attention not only for their intrinsic antimicrobial properties but also for their pleiotropic effects, including anti-inflammatory, anti-cancer, and immunomodulatory activities [32,33,34]. Derived from a vast array of plant species, these compounds often exhibit favorable safety profiles and a broad spectrum of biological activities. Critically, when co-administered with conventional antibiotics, many plant-derived natural products have been shown to significantly enhance bacterial growth inhibition and impede microbial proliferation [35,36,37,38,39]. Their utility as antibiotic adjuvants offers a compelling strategy to potentiate antimicrobial efficacy, enabling the reduction in antibiotic dosages, thereby mitigating side effects and, crucially, slowing the development of drug resistance. The judicious integration of phytochemicals into antibiotic regimens thus holds immense promise for advancing human health and refining contemporary therapeutic strategies.

Among phytochemicals, alkaloids, a vast class of nitrogen-containing compounds, are widely distributed and structurally diverse [40,41]. Characterized by complex heterocyclic rings, these molecules are fundamental to plant physiology and represent a significant pool for drug discovery [42,43]. A growing body of research highlights their potential as potent antibiotic adjuvants [44]. Berberine (BER), an isoquinoline quaternary alkaloid widely isolated from medicinal plants such as *Coptis chinensis* and *Phellodendron amurense* [45,46], exemplifies such potential. It acts as a potent antibiotic adjuvant, broadly enhancing the efficacy of diverse antibiotic classes, including β-lactams, quinolones, aminoglycosides, tetracyclines, and macrolides, against drug-resistant bacteria [47,48,49,50]. This potentiation often manifests as a significant reduction in the minimum inhibitory concentration (MIC) and, notably, can reverse bacterial resistance to susceptibility. For instance, Zhou et al. [51] demonstrated that BER synergistically enhanced ciprofloxacin’s activity against multidrug-resistant (MDR) *Klebsiella pneumoniae*, reducing MICs and exhibiting combined synergistic (18.18%) and additive (77.27%) effects. Mechanistically, this synergy was linked to the suppression of acrAB-tolC and acrR multidrug efflux pump gene expression. Similarly, Shi et al. [52] found BER-ciprofloxacin synergy against Salmonella biofilm formation, mediated by repressing genes critical for biofilm development (*luxS*, *rpoE*, *ompR*). Furthermore, Fu et al. [53] reported that BER significantly lowered the MICs of ampicillin and oxacillin against MRSA, achieving additive and synergistic effects, respectively. Beyond direct antimicrobial potentiation, BER also reduced MRSA adhesion and inhibited intracellular invasion in human gingival fibroblasts, suggesting multi-modal adjuvant properties. Another promising alkaloid, matrine, a nitrogen-containing heterocyclic compound derived from *Sophora flavescens* [54], also possesses diverse pharmacological activities, including antibacterial and anti-inflammatory effects. While matrine derivatives themselves can exhibit potent antibacterial activity, surpassing conventional antibiotics like penicillin G and fluconazole in some cases, with specific structural features (e.g., electron-withdrawing groups) identified as key for enhanced activity through 3D-QARS modeling [55], its adjuvant potential is particularly compelling. When combined with antibiotics, matrine effectively disrupts bacterial biofilm integrity and significantly reduces MICs. Pourahmad et al. [56] showed that matrine strongly inhibited biofilm formation in MDR *Pseudomonas aeruginosa*, *Escherichia coli*, and *Staphylococcus epidermidis*. Furthermore, it suppressed the expression of AcrAB-TolC efflux pump genes in *E. coli*, thereby reversing ciprofloxacin resistance when used in combination.

Polyphenols, a vast group of plant metabolites, are renowned for their diverse biological activities, including antioxidative, anti-inflammatory, and anticancer effects, alongside their direct antibacterial properties [57,58]. Structurally categorized into flavonoids (e.g., *flavonols*, *flavones*, *flavanols*) and non-flavonoids (e.g., benzoic acid derivatives, cinnamic acid derivatives, stilbenes), these compounds represent another significant class of phytochemicals with adjuvant potential. For instance, tea polyphenols, in combination with various antibiotics (e.g., imipenem, piperacillin), exhibited synergistic bactericidal effects against MDR Klebsiella pneumoniae, primarily by disrupting bacterial outer membrane formation and inhibiting extracellular polymeric substance production [59]. The synergistic potential extends to stilbenes, as Li et al. [60] demonstrated that a combination of benzyl isothiocyanate and resveratrol significantly enhanced antimicrobial activity against Listeria monocytogenes. This synergy involved suppressing bacterial motility, reducing biofilm formation by 56.4%, disrupting cell membranes, and inducing intracellular reactive oxygen species (ROS) surges. Another notable polyphenol, epigallocatechin gallate (EGCG) from green tea, and its modified derivative EGCG-stearate, have shown promising anti-biofilm activity and synergistic effects with antibiotics against a range of pathogenic bacteria, including *E. coli*, *Pseudomonas aeruginosa*, and *Staphylococcus aureus* [61]. Mechanistically, flavonoids such as α-mangostin (AMG) and isobavachalcone (IBC) not only exert rapid bactericidal effects against Gram-positive bacteria but also restore colistin susceptibility in Gram-negative pathogens. Their mechanism involves interacting with bacterial membrane phospholipids, leading to proton motive force (PMF) dissipation and metabolic disturbances [62]. Furthermore, caffeic acid has been shown to broaden the efficacy of antibiotics like erythromycin, clindamycin, cefoxitin, and vancomycin against *S. aureus* isolates when incorporated at sub-inhibitory concentrations [63].

Essential oils (EOs), complex natural blends comprising over a hundred volatile constituents, primarily terpenes and terpenoids along with other low molecular weight aromatic and aliphatic compounds [64,65], also represent a potent source of adjuvants. EOs are well-documented for their broad pharmacological activities, including significant antifungal, antibacterial, and antiviral properties, making them attractive candidates for novel antimicrobial strategies in medicine and food preservation [66]. A key mechanism underlying their antimicrobial action, and thus their synergistic potential with antibiotics, involves the disruption of bacterial cell walls and membranes [67]. This synergistic interaction offers a promising avenue for combating AMR. For example, Oliveira et al. [68] reported synergistic effects when Croton conduplicatus essential oil (EOCC) was combined with oxacillin (OXA) and ampicillin (AMP). This combination drastically reduced the MICs of OXA and AMP for both methicillin-sensitive and methicillin-resistant *S. aureus* (MSSA/MRSA), and importantly, altered the resistance profile of these strains. EOCC further demonstrated partial inhibition of biofilm formation. Similarly, Litsea cubeba essential oil (LCEO) inhibited the growth and facilitated the eradication of Vibrio parahaemolyticus biofilms by reducing hydrophobic components and extracellular polysaccharides on the cell surface when co-administered with antimicrobial drugs [69].

#### 2.1.2. Natural Products Derived from Animals

Beyond botanical sources, the animal kingdom offers a diverse array of natural products, typically serving as metabolic byproducts or endogenous compounds crucial for physiological functions [70,71]. Recent research has increasingly focused on the antibiotic synergistic effects exhibited by these animal-sourced molecules. For instance, L-lysine, an exogenous amino acid, was found to enhance the susceptibility of Acinetobacter baumannii, other Gram-negative bacteria, and even Gram-positive Mycobacterium smegmatis to aminoglycosides. Deng et al. [72] demonstrated that combining L-lysine with aminoglycosides effectively eliminated clinical MDR A. baumannii strains, including challenging persister cells. Mechanistically, L-lysine augments the proton motive force (PMF) via transmembrane chemical gradient modulation, thereby increasing aminoglycoside accumulation and contributing to reactive oxygen species generation. Melatonin, a neurohormone, has also emerged as an antibiotic adjuvant. Miro-Canturri et al. [73] reported that LPC administration reduced bacterial load in tissues and blood, significantly improving survival rates in mice with peritoneal sepsis.

**Figure 2:**
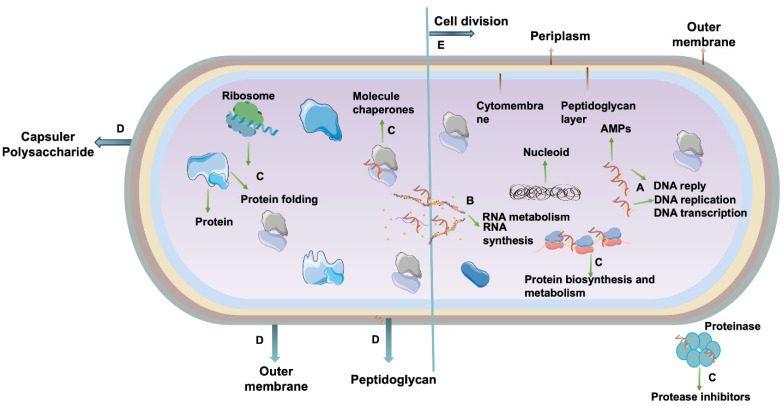
**Physiological processes and targets of AMPs in drug-resistant bacteria.** (A) DNA-related physiological regulation, such as DNA stress response, transcription, and replication; (B) RNA synthesis and metabolic intervention, involving the regulation of RNA synthesis pathways and metabolic activities; (C) protein synthesis and folding regulation, affecting protein biosynthesis, folding processes, and protease activity modulation; (D) bacterial outer membrane and cell wall targeting, acting on bacterial outer membrane structures or cell wall peptidoglycan components; (E) bacterial division process interference, inhibiting the normal physiological progression of bacterial cell division.

Antimicrobial peptides (AMPs), key effectors of the host’s innate immune system, are peptide-based bioactive substances exhibiting broad-spectrum inhibitory activity against viruses, bacteria, fungi, and parasites (Figure 2). While many AMPs possess direct antimicrobial activity, some demonstrate significant adjuvant potential. For example, SLAP-S25, a synthetic short linear antibacterial peptide, despite modest intrinsic activity, markedly potentiated the efficacy of diverse antibiotic classes (e.g., cefepime, colistin, ofloxacin, vancomycin) against MDR Gram-negative pathogens [74]. In another compelling example, Wang et al. [75] unveiled a novel antimicrobial strategy combining Nisin (NIS), a bacteriocin from Lactococcus lactis, with oxacillin against MRSA. Through comprehensive in vitro and in vivo studies, NIS was shown to restore oxacillin sensitivity by suppressing mecA transcription, inhibiting biofilm formation, and inducing cellular morphological alterations. This combinatorial approach significantly attenuated MRSA virulence in a murine skin infection model, positioning NIS as a promising therapeutic adjuvant against antibiotic-resistant pathogens.

#### 2.1.3. Microbial-Derived Natural Products

Beyond plant and animal sources, microorganisms themselves can yield natural products or insights that lead to potent adjuvants, often by targeting bacterial metabolism. Metabolic reprogramming has emerged as a sophisticated and highly effective strategy for modulating bacterial antibiotic susceptibility [76]. Pioneering work by Allison et al. [77] demonstrated that exogenous metabolites, including glucose, mannitol, and fructose, could dramatically enhance aminoglycoside efficacy against drug-resistant *E. coli* and *S*. *aureus*, achieving over a 1000-fold increase in bactericidal activity. This metabolite-mediated potentiation operates by augmenting cellular antibiotic uptake through the activation of the electron transport chain and enhancement of the PMF, thereby offering a fundamental approach to circumvent bacterial antibiotic resistance.

The broad applicability of metabolic modulation as a strategy to overcome bacterial antibiotic resistance across diverse microbial species is increasingly evident [78,79]. Peng et al. [80] further illustrated this by showing that exogenous metabolites like alanine and glucose could restore antibiotic susceptibility in MDR Edwardsiella tarda. This was achieved by enhancing tricarboxylic acid (TCA) cycle activity, increasing NADH production, and strengthening the PMF. Notably, this approach exhibited remarkable cross-species efficacy, potentiating antibiotic absorption in both Gram-negative (*Vibrio parahaemolyticus*, *Klebsiella pneumoniae*, *Pseudomonas aeruginosa*) and Gram-positive (*Staphylococcus aureus*) bacteria. Furthermore, these metabolite-antibiotic synergies extended beyond aminoglycosides to quinolones, effectively overcoming bacterial tolerance during the stationary phase by reactivating respiratory metabolism. Such findings strongly advocate for a transformative, metabolomics-driven approach to reverse antibiotic resistance through strategic metabolic reprogramming.

### 2.2. Artificially Synthesized Molecules

Artificially synthesized molecules are rapidly gaining recognition as indispensable components in advanced antibiotic therapies, holding immense potential to significantly amplify the efficacy of existing antimicrobial agents and expand their operational spectra against increasingly resistant pathogens [81,82,83]. This domain encompasses a diverse array of precisely engineered compounds, ranging from nanomaterials to synthetic peptides, all designed to circumvent or disable bacterial resistance mechanisms [84,85]. The strategic development of these innovative antibiotic adjuvants paves the way for substantial improvements in clinical outcomes [85,86]. Illustrating this potential, Ferreres et al. [87] engineered novel, multifunctional nanocomposites by integrating antimicrobial silver nanoparticles (NPs), biocompatible chitosan, and acylase I—an enzyme adept at degrading bacterial quorum sensing signal molecules. This sophisticated design demonstrated a synergistic effect: the acylase-conjugated NPs significantly reduced P. aeruginosa biofilm formation by approximately 55%, while the silver/chitosan framework effectively compromised bacterial membrane integrity, leading to the complete eradication of planktonic cells.

In parallel, targeted inhibition of specific resistance mechanisms has yielded promising synthetic adjuvants. Gao et al. [88] developed a potent N-acylhydrazone scaffold that effectively inhibits metallo-β-lactamases (MBLs), critical enzymes conferring broad-spectrum resistance. Their series of compounds exhibited high selectivity for NDM-1, with compound 1 demonstrating exceptional inhibitory activity (IC50 of 1.2 μM). Structure-activity relationship (SAR) analysis revealed that strategic incorporation of pyridine and hydroxylbenzene substituents at the 2-position significantly enhanced NDM-1 inhibition. Enzymatic kinetics confirmed that compound 1 functions as a competitive and reversible NDM-1 inhibitor. Critically, these synthesized compounds exhibited synergistic antibacterial activity with meropenem, leading to a substantial 4- to 16-fold reduction in MIC against NDM-1-producing *E. coli* BL21 (DE3).

Further expanding the repertoire of MBL inhibitors, Liu et al. [89] synthesized a series of 25 thiosemicarbazones, systematically evaluating their inhibitory effects against MBLs such as ImiS and NDM-1. Compound 2a stood out for its dual action: it not only exhibited potent antibacterial activity against various clinical isolates (e.g., K. pneumoniae-KPC-NDM) with MICs ranging from 16 to 128 μg/mL, but also demonstrated remarkable synergy with meropenem, reducing the MICs by factors of 8 to 32 μg/mL against the tested resistant strains.

Beyond direct enzyme inhibition, synthetic peptides offer a distinct approach. Ramirez et al. [90] designed dUSTBP8, a novel lipopeptide composed of three L-arginine residues and a double eight-carbon lipid moiety. This compound exhibited highly desirable properties, including a lack of hemolytic activity and remarkable stability in the presence of pancreatic enzymes. When co-administered with conventional antibiotics, dUSTBP8 displayed potent synergistic antibacterial effects against Gram-negative bacteria, particularly Acinetobacter baumannii. Specifically, it enhanced the antibacterial efficacy of levofloxacin by 16- to 64-fold and neomycin by an impressive 32- to 512-fold, culminating in the complete eradication of bacteria within 24 h. These examples underscore the profound impact of rational design and synthetic chemistry in creating tailored adjuvants to restore antibiotic potency.

### 2.3. Bacteriophages: Orchestrating a Synergistic Attack

**Figure 3:**
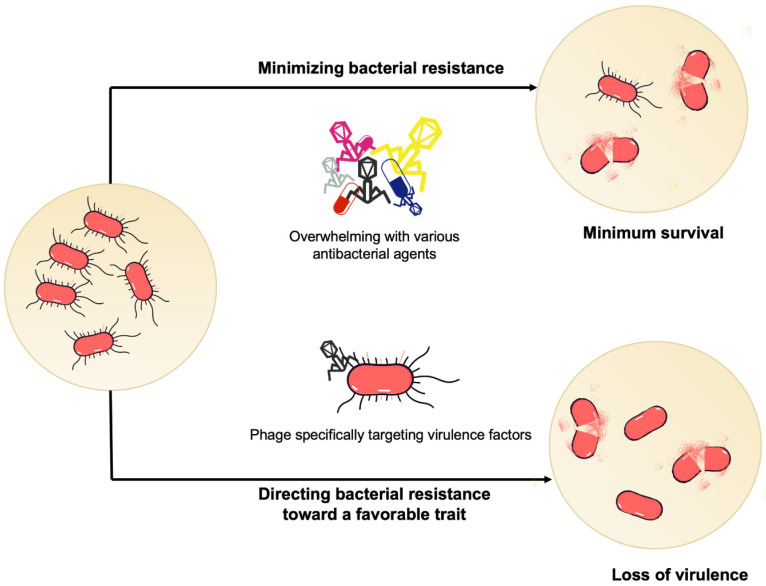
**Strategies for Mitigating Bacterial Resistance in Phage Therapy.** Strategy 1: Suppression of Bacterial Resistance Emergence. By combining multiple therapeutic modalities, this strategy synergistically eliminates diverse genotypes of target bacteria, thereby reducing bacterial population size and weakening their capacity to evolve resistance. Strategy 2: Guiding Resistance Toward Benign Evolution. Using therapeutic phages that specifically bind to bacterial virulence factors, selective pressure is applied to induce bacteria to delete or modify virulence-associated structures. This drives the survival of resistant bacterial populations toward low-virulence or avirulent phenotypes.

Bacteriophage-based antimicrobial strategies represent a highly sophisticated and rapidly evolving approach to counteracting the escalating crisis of bacterial resistance [91]. Recent investigations have unveiled the remarkable potential of combinatorial antimicrobial interventions, leveraging bacteriophages not merely as direct lytic agents but as powerful adjuvants that orchestrate intricate mechanisms of bacterial control. Innovative research has particularly highlighted the synergistic potential when bacteriophages are judiciously combined with conventional antibiotics (Figure 3). A groundbreaking study by Liu et al. [92] revealed that the concurrent administration of the antiseptic chlorhexidine gluconate (CHG) and bacteriophage vB_3530 achieved unprecedented suppression of CHG-resistant *Pseudomonas aeruginosa*. This combination not only led to rapid bacterial elimination but also crucially prevented the emergence of further resistance. Complementary research by Song et al. [93] characterized the lytic bacteriophage vB_1086, demonstrating its potent antibacterial synergy with ceftriaxone and its remarkable capacity to inhibit biofilm formation in combination with meropenem. Despite these promising advancements, significant challenges persist in optimizing phage-antibiotic pairings. It is also important to acknowledge that phage-antimicrobial interactions are not universally synergistic; depending on the specific phage, the targeted bacterial strain, and the sequence of administration, interactions can occasionally be neutral or even antagonistic, underscoring the need for precision in combinatorial design. The current paradigm often remains empirical, fraught with uncertainties regarding the precise selection of optimal phage strains, and the complex host-specific modulating factors that influence combinatorial efficacy. The intricate molecular interplay between bacteriophages and bacterial systems necessitates far more nuanced and mechanistic investigations. Nevertheless, the burgeoning field of bacteriophage research stands as a pivotal frontier in antimicrobial strategy, simultaneously offering innovative solutions and illuminating the profound complexity of bacterial-phage interactions. Future research must rigorously address the multifaceted challenges inherent in developing predictable, targeted antimicrobial interventions that can effectively mitigate bacterial resistance while judiciously minimizing unintended ecological consequences.

### 2.4. Clinical Therapeutic Drugs: Repurposing for Resistance

Beyond their established primary therapeutic applications, a growing number of clinical drugs are being judiciously repurposed to serve as potent antibiotic adjuvants, thereby significantly broadening their pharmaceutical utility in the fight against antimicrobial resistance (AMR) [94]. This innovative strategy leverages existing pharmacokinetic and safety profiles, accelerating the translation of novel adjuvant concepts. A compelling example is N-acetylcysteine (NAC), traditionally recognized for its mucolytic and antioxidant properties as an expectorant [95]. Groundbreaking research by Pollini et al. [96] revealed NAC’s remarkable capacity to dramatically reverse the colistin-resistant phenotype of Acinetobacter baumannii. Their investigation unveiled a nuanced efficacy profile, demonstrating complete bactericidal effects against resistant strains within 24 h in a dose-dependent manner. Intriguingly, NAC exhibited markedly limited efficacy against colistin-susceptible bacterial strains, suggesting a specific interaction with resistance mechanisms rather than broad-spectrum intrinsic antimicrobial activity—an important aspect for minimizing selective pressure and preserving the microbiome.

Further expanding the paradigm of drug repurposing, the investigation by Jiang et al. [97] highlighted metformin—a cornerstone in diabetes management—as a potent antibiotic enhancer. This research compellingly demonstrated metformin’s ability to augment tetracycline efficacy against a broad spectrum of multidrug-resistant pathogens, including Staphylococcus aureus, Enterococcus faecalis, *E. coli*, and Salmonella enteritidis. Mechanistically, this enhancement was elucidated to involve increased intracellular doxycycline accumulation in tetracycline-resistant *E. coli*, thereby effectively circumventing established efflux-mediated resistance mechanisms.

Building upon these innovative approaches, the research by Ayerbe-Algaba et al. [98] elucidated a sophisticated mechanism for oxyclozanide-a veterinary antiparasitic agent-in disrupting bacterial membranes. When co-administered with colistin, oxyclozanide exhibited a unique capacity to augment antibiotic efficacy against notoriously challenging Gram-negative pathogens, including Burkholderia cepacia, *Pseudomonas aeruginosa*, and Klebsiella pneumoniae. The compound’s targeted, localized membrane disruption appears to enhance colistin binding and uptake, offering a novel and effective strategy to overcome intrinsic and acquired bacterial resistance to this last-resort antibiotic.

## 3. Antibiotic Adjuvant Mechanisms of Action

Antibiotic adjuvants exert their therapeutic effects through a diverse array of molecular mechanisms, often targeting critical bacterial vulnerabilities that enable resistance or resilience.

### 3.1. Enzyme Inhibition as a Targeting Strategy Against Resistance

Enzymatic inactivation constitutes a pervasive and formidable mechanism of antimicrobial resistance, whereby bacteria deploy a diverse array of enzymes capable of degrading or covalently modifying antibiotic compounds, thereby compromising their therapeutic efficacy [99,100,101]. The arsenal of resistance enzymes identified to date encompasses an extensive range of mechanisms, targeting virtually all major antibiotic classes, including β-lactams, aminoglycosides, phenicols, and macrolides [29,102,103,104]. Consequently, a highly promising therapeutic strategy involves the development of enzyme inhibitors that can restore antibiotic susceptibility by preventing the enzymatic deactivation of antimicrobial compounds [105]. The remarkable clinical success of β-lactamase inhibitors stands as a prime example of this approach. These inhibitors are mechanistically categorized into two principal classes: irreversible and reversible. Irreversible inhibitors, exemplified by clavulanic acid, sulbactam, and tazobactam, typically incorporate β-lactam-like scaffolds or appropriate steroidal structures that target the active site or proximal regions of β-lactamases, leading to permanent enzyme inactivation [106]. This strategy has culminated in several clinically approved antibiotic-inhibitor combinations, such as amoxicillin-clavulanic acid (e.g., Augmentin), ticarcillin-clavulanic acid, ampicillin-sulbactam, and piperacillin-tazobactam [88]. Clavulanic acid, for instance, forms an irreversible covalent adduct with the catalytic serine residue ofβ-lactamases, thereby precluding the hydrolysis of co-administered amoxicillin [107]. In contrast, reversible inhibitors, such as the more recently developed avibactam and vaborbactam, form transient associations with their target enzymes. While highly effective, their inhibitory effects may be attenuated through dilution or competitive displacement [108]. The continued innovation in β-lactamase inhibitor development remains a cornerstone strategy in antibiotic adjuvant research.

Recent advances have expanded this enzyme inhibition paradigm beyond β-lactamases to target other critical resistance mechanisms. Notable progress has been made in developing tetracycline derivatives that counter specific resistance pathways. For instance, anhydrotetracycline and its analogues demonstrate significant inhibitory activity against the Tet(X7) enzyme in clinical isolates of *Pseudomonas aeruginosa* through both competitive and non-competitive mechanisms, effectively restoring tetracycline sensitivity in otherwise resistant strains [109]. Furthermore, emerging research has identified protein folding pathways as promising therapeutic targets, given that the functionality of many antibiotic resistance enzymes critically depends on their proper protein conformation. A compelling example is the targeting of DsbA, a key disulfide bond oxidoreductase. Disruption of DsbA function leads to β-lactamase inactivation and destabilization of mobile colistin resistance enzymes. Significantly, DsbA inhibition has been shown to restore antibiotic susceptibility in multidrug-resistant (MDR) clinical isolates and enhance survival in Galleria mellonella infection models challenged with MDR P. aeruginosa, highlighting the therapeutic potential of modulating protein folding pathways in combating AMR.

### 3.2. Suppressing the Activity of Efflux Pumps

Efflux pumps represent a formidable defense mechanism employed by drug-resistant pathogens, actively extruding a wide array of antibiotics from bacterial cells and thereby preventing their intracellular accumulation to therapeutic concentrations [110]. This active efflux is a pivotal factor contributing to antibiotic treatment failure. Current strategies for developing antibiotic adjuvants primarily impact efflux pump activity through two distinct mechanisms: direct structural disruption of the efflux pump complex or downregulation of efflux pump gene expression [111]. Dwivedi et al. [112] identified niaziridin, niazirin (derived from Moringa oleifera pods), and ouabain as potential agents for reversing drug resistance. These compounds demonstrate efficacy by inhibiting efflux pumps and modulating the expression patterns of genes associated with drug resistance. Notably, niaziridin effectively downregulates two critical efflux pump genes, acrB and yojI, both individually and in combination. Additionally, niaziridin was observed to enhance the expression of porin-forming genes (ompA and ompX), further contributing to antibiotic uptake. Salaheen et al. [112] investigated phenolic extracts from blueberry and blackberry pomace (BPE) against MRSA. They discovered that BPE, rich in chlorogenic, ferulic, vanillic, caffeic, and ellagic acids, completely inhibited MRSA growth in vitro. Further investigation revealed that BPE restored MRSA susceptibility to methicillin by suppressing the expression of key efflux pump genes (norA, norB, norC, mdeA, sdrM, sepA) in Staphylococcus aureus.

Critically, bacterial efflux pumps typically harness the electrochemical energy embedded within the proton motive force (PMF) as their propulsive mechanism [113]. Consequently, pharmaceutical agents that disrupt the PMF can serve as potent antibiotic adjuvants by impeding the functionality of these pumps through energetic starvation. This, in turn, facilitates the intracellular accumulation of antibiotics and reinstates their bactericidal efficacy against resistant strains.

### 3.3. Alterations in Bacterial Membrane Permeability

The therapeutic efficacy of antimicrobial agents is fundamentally contingent upon their ability to traverse bacterial cell membranes and reach their intracellular targets [114]. Membrane permeability critically influences antibiotic penetration rates, with distinct mechanisms governing the entry of hydrophobic and hydrophilic compounds [115]. While hydrophobic antibiotics can directly diffuse through the lipid bilayer, hydrophilic compounds typically rely on membrane-spanning porins for cellular entry [116]. This dichotomy underscores how membrane composition, lipid organization, and porin characteristics collectively modulate bacterial susceptibility to antimicrobial agents.

Antibiotic adjuvant development have revealed several promising strategies to enhance antimicrobial uptake by strategically modulating membrane properties [117,118]. One approach involves the manipulation of bacterial membrane potential (ΔΨ) and transmembrane proton gradients to overcome intrinsic resistance mechanisms [119]. A notable example is the application of cerium oxide (CeO_2_) nanoparticles, which Bellio et al. [120] demonstrated can selectively interact with and increase the permeability of Gram-negative bacterial membranes without compromising host cell integrity. This property, combined with their low cytotoxicity profile, positions CeO_2_ nanoparticles as promising adjuvants for combating multidrug-resistant pathogens.

Membrane permeabilization can also be achieved through various stress-inducing agents that target specific membrane components. Ellis et al. [121] elucidated how magnesium ion depletion enhances outer membrane permeability by disrupting the stabilizing interactions between divalent cations and negatively charged lipopolysaccharides (LPS). Similarly, Corbett et al. [122] demonstrated that SPR741, an outer membrane protein inhibitor, dramatically potentiates various antibiotics, achieving up to 8000-fold reductions in MICs. However, this enhancement was less pronounced against strains where resistance was primarily mediated by efflux pump systems, highlighting the specificity of its action. Other membrane-targeting agents, including chelators such as EDTA and antimicrobial peptides like polymyxin, function by disrupting the cationic bridges between LPS molecules, thereby compromising outer membrane integrity [123].

Genetic approaches to manipulating membrane permeability have provided additional insights into potential therapeutic strategies. Klobucar et al. [124] demonstrated that mutations affecting outer membrane-associated proteins, particularly those involved in LPS core biosynthesis, can lead to increased phospholipid exposure on the outer leaflet of bacterial outer membranes. This altered membrane architecture enhances the effectiveness of hydrophobic antibiotics, suggesting that targeted manipulation of membrane protein expression could represent a viable strategy for antibiotic potentiation. These findings collectively underscore the profound potential of membrane-targeted approaches in developing more effective antimicrobial therapies against resistant pathogens.

### 3.4. Metabolic Reprogramming

**Figure 4:**
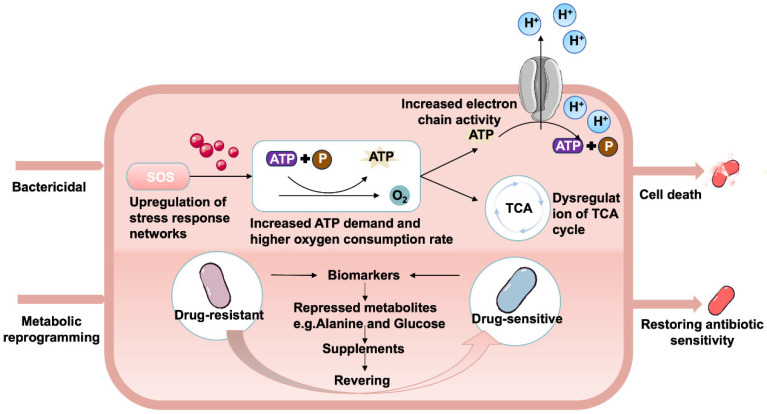
**Antibiotic adjuvants mediate bacterial metabolic reprogramming to kill bacteria.** The upper cell diagram shows that bactericidal antibiotics induce cell death through multiple mechanisms. Following initial target inhibition, they primarily stimulate cellular metabolism, including upregulating stress response networks, electron transport chains, and tricarboxylic acid cycle (TCA) activity. The lower part shows that under the action of exogenous alanine and/or glucose, the susceptibility of drug-resistant bacteria to antibiotics can be restored by increasing tricarboxylic acid cycle (TCA) flux, NADH production, and proton motive force.

The strategic reprogramming of the bacterial metabolome by exogenous compounds, often challenging to achieve through traditional genetic methods, is facilitated by leveraging distinct metabolic pathways and shared metabolites among species. The metabolome represents a comprehensive collection of cellular end products that reflect the cellular state at a specific stage [125,126]. Specific metabolite provision can sensitize cells resistant to certain factors, making metabolomics approaches invaluable not only for disease biomarker screening but also for identifying key metabolites or potential drugs to restore or enhance sensitivity as required (Figure 4) [127,128,129]. Recently, the intricate interplay between various types of metabolites and transport proteins/receptor proteins with hormones, fatty acids, drugs, or other exogenous substances has garnered significant attention due to its profound impact on protein conformation and function [130,131,132].

Exogenous key metabolites can be strategically utilized to reprogram the bacterial metabolic network, thereby generating specific metabolic profiles that adapt to changes in both internal and external environments. By altering bacterial metabolism, the susceptibility of antibiotics can be profoundly influenced. This strategy was first pioneeringly implemented over a decade ago by Allison et al. [77], aiming to enhance the efficacy of aminoglycoside drugs against resistant *E. coli* and Staphylococcus aureus. Peng et al. [80] subsequently found that combining kanamycin with key metabolites from glycolysis (such as glucose, mannitol, and fructose) could yield over 1000-fold greater bacterial killing compared to kanamycin alone. These metabolites increase PMF by activating the electron transport chain, thereby enhancing the uptake of aminoglycoside antibiotics and their intracellular concentration. This metabolism-dependent antibiotic killing enhancement strategy has also been successfully applied to bacteria intrinsically resistant to aminoglycoside antibiotics.

Metabolites play a pivotal role in cellular activity, serving as both substrates for enzymes and regulators of other molecules, including proteins and nucleic acids. The abundance of specific metabolites is crucial for their biological function, as it governs the activity of enzymes and the actions of regulated molecules, thereby influencing metabolic pathways and networks and potentially modulating metabolic flux [133,134,135]. Differential activation or inhibition of one or more metabolic pathways may serve as a key characteristic of phenotypic responses. If the reactive “metabolic group” can be identified, exogenous administration of specific critical metabolites from these groups could induce either sensitivity or resistance to reaction-induced therapy [136]. In essence, by engineering modifications to the metabolic environment, responsive or resistant states can be achieved by transforming susceptible or resistant metabolic groups, respectively, leading to a profound reprogramming of the metabolome. Reprogramming metabolomics approaches can thus elucidate mechanisms underlying cellular responses, facilitating the development of convenient and effective methods for controlling and/or preventing pathogen infections while simultaneously enhancing host immunity [137,138].

### 3.5. Signaling Inhibitors

**Figure 5:**
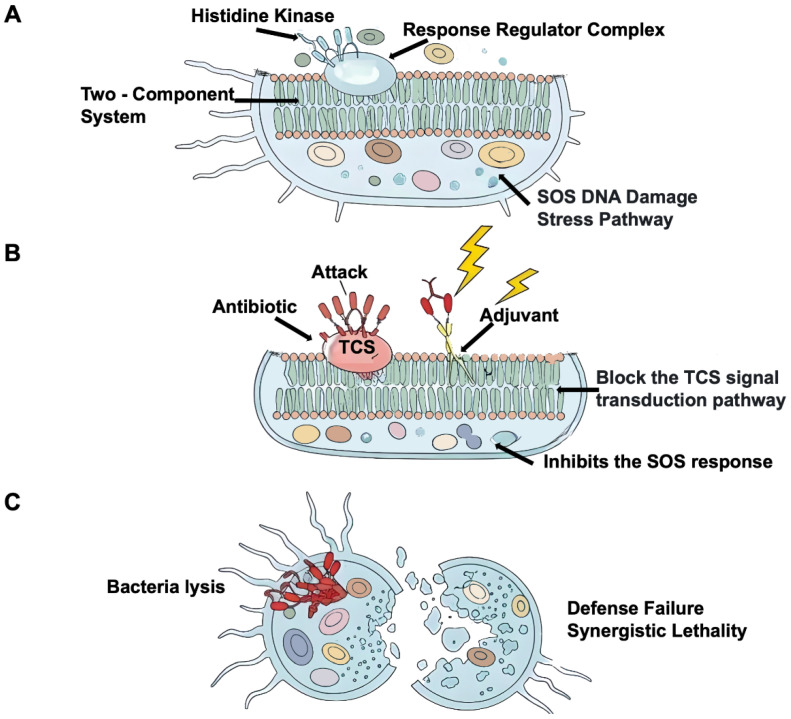
**Antibiotic adjuvants exert bactericidal or bacteriostatic effects through signal inhibition.** (**A**) TCS is distributed on the cell membrane and collaborates with the intracellular SOS DNA damage stress pathway to form the molecular basis of bacterial environmental perception and defense regulation. (**B**) Antibiotics act directly on bacteria, while adjuvants target and interfere with the TCS signal transduction cascade and inhibit key nodes of the SOS pathway (RecA/LexA complex), synergistically disrupting the bacterial stress defense network. (**C**) Due to the dual blockade of TCS-mediated environmental perception and the DNA damage repair function of the SOS pathway, the bacterial defense system collapses, leading to cell lysis.

Bacterial signal transduction critically relies on two-component signal transduction systems (TCSs), which serve as the primary mechanism for bacteria to perceive and respond to various environmental changes during growth and reproduction. These systems are crucial for adaptation, spore production, and toxin generation (Figure 5) [139,140,141]. Bacterial TCSs typically consist of a histidine kinase and a cognate response regulator. The histidine kinase perceives external signals and consequently modifies the phosphorylation state of conserved amino acid residues within the bacterial cell, which then activates the response regulator to modulate gene transcription or modify other proteins for fundamental signal transduction processes. Martin et al. [142] notably demonstrated that in Staphylococcus aureus, specific TCSs encoded by yycFG genes, when point mutated, render bacteria significantly more susceptible to antibiotic-induced killing. Moreover, inhibition of these TCSs by small molecules has been shown to enhance host-mediated bacterial clearance.

Bacteria are perpetually exposed to a myriad of adverse environments, with pathogenic bacteria enduring ceaseless onslaughts from physical and chemical factors that inflict damage upon their DNA [143]. In response, bacteria have ingeniously developed intricate mechanisms to uphold and mend their genetic material, exemplified by the remarkable SOS response [144,145]. This extraordinary biological defense system not only encompasses the repair of DNA lesions but also confers antibiotic resistance and facilitates horizontal gene transfer. Pioneering investigations conducted by Memar et al. [146] have unequivocally demonstrated that impeding the bacterial SOS system can significantly diminish the MIC of specific antibiotics. Targeting the core defense proteins RecA and LexA effectively neutralizes the bacterium’s primary survival mechanism, restoring its susceptibility to antibiotics [147]. For instance, sulamine exhibits potency through inhibition of ATPase activity and DNA strand exchange reactions; N6-(1-naphthyl)-ADP effectively curbs RecA function; the PSiB protein encoded by plasmid R6-5 acts as an impediment against LexA cleavage to suppress the SOS response; while various metabolites derived from lichens serve as potent RecA inhibitors. These exceptional substances act as superb adjuncts to antibiotics, augmenting their antibacterial efficacy by disarming bacterial stress responses.

### 3.6. Biofilms

Bacterial biofilms represent complex, three-dimensional communities encased in an extracellular matrix. While they do not inherently alter the MIC of antibiotics against individual planktonic bacteria, they create a formidable barrier to antimicrobial therapy through multiple, synergistic mechanisms [148,149,150]. These structured communities impose physical restrictions on antibiotic penetration, acting as a diffusion barrier, and significantly modulate bacterial metabolic states, resulting in drastically reduced growth rates and altered physiological profiles within the biofilm matrix [151]. This metabolic adaptation, often coupled with increased osmotic pressure, leads to the downregulation of porin expression, further limiting antibiotic access to bacterial cells and promoting the development of antimicrobial resistance [152]. Moreover, biofilms create optimal microenvironments for horizontal gene transfer, facilitating the rapid dissemination of resistance determinants among bacterial populations and contributing significantly to therapeutic failure and the persistence of chronic infections [153].

Recent advances in antibiotic adjuvant development have therefore focused on enhancing antimicrobial penetration through biofilm matrices as a crucial therapeutic strategy. A notable innovation involves the covalent modification of antibiotics with nitrogen oxide (NO) moieties, which significantly improves their ability to penetrate biofilms and access bacterial cells, resulting in effective eradication of established Staphylococcus aureus biofilms. This approach has been further refined through the incorporation of NO-donating groups into the core structure of antimicrobial agents, as demonstrated with isothiazolone derivatives. These modified compounds exhibit reduced human cell cytotoxicity while maintaining potent anti-biofilm activity, capable of independently disrupting established bacterial biofilms [154]. An alternative, yet complementary, therapeutic approach involves the development of biofilm-dispersing agents that function as antibiotic adjuvants. These compounds disrupt the complex architecture of bacterial communities, converting aggregated, protected populations into more susceptible planktonic cells. This strategy effectively circumvents the protective mechanisms conferred by the biofilm structure, offering a crucial solution to the challenging problem of biofilm-associated infections. The strategic combination of biofilm-dispersing agents with conventional antibiotics represents a promising and impactful avenue for treating persistent infections that have traditionally proven recalcitrant to standard antimicrobial therapy [155].

## 4. Evolving Screening Methodologies for Antibiotic Adjuvants

The escalating crisis of antimicrobial resistance (AMR) necessitates the rapid and efficient discovery of novel antibiotic adjuvants. This imperative has driven significant advancements in screening methodologies, ranging from refined traditional approaches to cutting-edge high-throughput and computational techniques, all aimed at identifying compounds that can restore or augment antibiotic efficacy.

### 4.1. Traditional Screening Methods

Traditional screening methodologies for assessing antimicrobial interactions remain foundational in drug discovery, offering robust and reliable frameworks despite the advent of high-throughput technologies. The checkerboard dilution method, a cornerstone approach, systematically evaluates drug combinations by calculating fractional inhibitory concentration indices (FICI) [156,157]. While this method exhibits remarkable reliability and utility in laboratory settings, the inherent challenges associated with standardized FICI interpretation have prompted the exploration of refined metrics, such as the fractional bactericidal concentration index (FBCI), which may offer enhanced reproducibility and clinical relevance [158]. Recent technological adaptations have further enhanced the utility of these classical methods. For instance, the checkerboard approach has successfully revealed notable synergistic interactions, exemplified by the combination of silver nitrate with Nisin against diverse bacterial strains, and the promising partnership between 6-gingerol and vancomycin in addressing vancomycin-resistant phenotypes mediated by efflux mechanisms [159]. These findings underscore the enduring relevance of the checkerboard method in identifying novel therapeutic strategies.

Time-kill kinetics studies complement checkerboard analyses by providing a critical temporal resolution of bactericidal effects. This dynamic approach proves particularly valuable in characterizing the rate and extent of bacterial killing in combination therapies, offering insights that static endpoints cannot capture [160]. Modern adaptations, incorporating automated cell counting and real-time monitoring systems, have significantly enhanced the precision and throughput of these studies. The agar diffusion method and E-test applications represent rapid screening approaches that have evolved to accommodate combination testing [161]. While these methods offer an intuitive visualization of drug interactions through zones of inhibition, their accurate interpretation demands careful consideration of diffusion kinetics and local concentration gradients. Recent innovations in imaging analysis and automation have improved the quantitative aspects of these techniques, although standardization across laboratories remains a significant challenge [162].

Contemporary developments have increasingly focused on integrating these traditional methods with computational approaches. Machine learning algorithms, for instance, are being increasingly employed to predict synergistic combinations based on historical screening data, thereby streamlining the discovery process [163]. Additionally, microfluidic adaptations of classical methods are emerging, offering the potential for increased throughput while maintaining the inherent robustness of traditional approaches.

Despite their established utility, these traditional methods face several limitations within the context of modern drug discovery. Their labor-intensive nature restricts their application in large-scale combination studies, and their in vitro nature may not fully recapitulate the intricate complexity of host–pathogen interactions [164,165]. Furthermore, the emergence of adaptive resistance mechanisms and the increasing recognition of bacterial persistence necessitate more sophisticated analytical frameworks that can capture dynamic bacterial responses [166,167]. The future evolution of traditional screening methods will likely involve their seamless integration with advanced technologies such as single-cell analysis and real-time monitoring systems [168,169]. Efforts to standardize interpretation criteria and establish robust correlations with clinical outcomes remain crucial challenges. Ultimately, the development of hybrid approaches that judiciously combine the reliability of traditional methods with the efficiency and depth of modern screening platforms offers a promising path forward in antimicrobial drug discovery [170].

### 4.2. Advanced Screening Techniques

**Figure 6:**
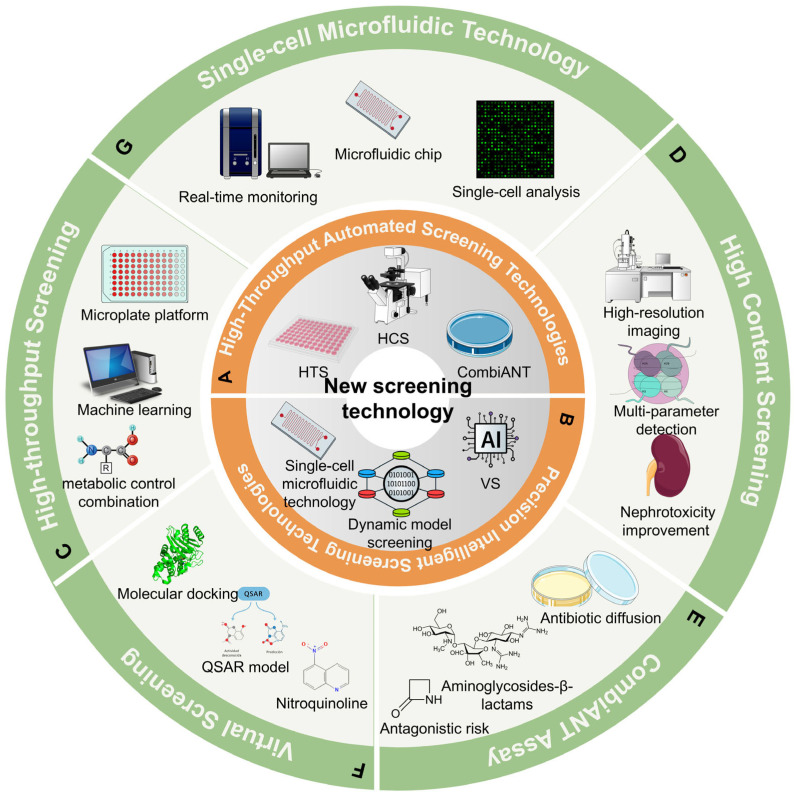
**Identifying new antibiotic adjuvants using novel screening technologies.** (**A**) High-Throughput Automated Screening Technologies: Focused on efficiency, large-scale technologies such as microplate platforms and agar diffusion are used for rapid primary screening of massive candidate compounds in the early stage, suitable for high-efficiency discovery of broad-spectrum adjuvants. (**B**) Precision Intelligent Screening Technologies: Oriented toward mechanistic analysis, computational simulations, microfluidic chips, and metabolic regulation models are employed to precisely identify adjuvant mechanisms, optimize molecular structures, and reveal antimicrobial heterogeneity at the single-cell level. (**C**) High-throughput screening (HTS) on microplate platforms uses machine learning to analyze thousands of compound combinations, rapidly identifying novel antibiotic adjuvants and metabolic regulation pairs. (**D**) High content screening (HCS) employs high-resolution imaging to monitor multi-parameters for identifying adjuvants with multiple mechanisms. (**E**) The CombiANT assay uses antibiotic diffusion on agar plates to evaluate synergies of three antibiotic pairs, identifying antagonistic risks in traditional combinations. (**F**) Virtual screening (VS) uses molecular docking and QSAR models to simulate compound-target binding, designing novel adjuvant structures. (**G**) Single-cell microfluidic technology isolates individual resistant bacteria in microchips to real-time monitor adjuvant-bacteria interactions.

The escalating challenge of antibiotic resistance has driven an urgent demand for innovative screening techniques capable of identifying effective adjuvants that can restore or enhance the efficacy of existing antibiotics. Advanced methodologies, including high-throughput screening (HTS), high-content screening (HCS) technology, phenotypic screening, and systems biology approaches, are collectively revolutionizing the discovery landscape for antibiotic adjuvants [171,172,173]. These techniques harness cutting-edge technologies such as automated liquid handling systems, microarray analysis, and sophisticated machine learning algorithms to rapidly and comprehensively assess vast libraries of compounds (Figure 6) [174,175,176,177].

#### 4.2.1. High-Throughput Screening (HTS)

HTS stands as a pivotal technological paradigm in contemporary drug discovery, enabling the comprehensive and expeditious evaluation of extensive compound libraries and diverse drug combination strategies. Leveraging microplate-based platforms, HTS facilitates parallel experimental processing, simultaneously interrogating millions of molecular interactions with unprecedented efficiency and precision. The technological sophistication of HTS relies on multifaceted molecular interrogation methodologies, encompassing intricate signal transduction pathway analysis, metabolic profiling, and detailed target site interaction characterization [178,179]. By systematically mapping complex pharmacological landscapes, researchers can strategically design rational drug combination interventions. For instance, the synergistic combination of Ibrutinib with CHEK1 inhibitors, validated through comprehensive genomic drug sensitivity consortium (GDSC) datasets, exemplifies HTS’s broader potential in elucidating targeted therapeutic approaches in drug discovery, including oncology [180]. In the domain of antimicrobial research, HTS has demonstrated remarkable potential for adjuvant discovery and combinatorial therapeutic strategies. Recent investigations have identified novel molecular candidates like tavaborole, which exhibits promising adjuvant properties against multidrug-resistant *E. coli*. Its mechanism potentially involves sophisticated interactions, including biofilm formation inhibition, membrane protein synthesis modulation, and enhancement of active transport processes [181]. Moreover, the convergence of HTS with advanced computational methodologies, particularly machine learning algorithms, has substantially expanded screening capabilities. Innovative studies have successfully engineered drug combinations incorporating D-amino acids with conventional antibiotics; notably, colistin combined with specific D-amino acid mixtures demonstrated significant in vivo efficacy against *Pseudomonas aeruginosa*, highlighting the transformative potential of integrated screening technologies [182]. However, a critical appraisal necessitates acknowledging HTS’s inherent methodological limitations. Despite its capacity for large-scale simultaneous sample processing, the technology remains susceptible to potential screening artifacts. Variability in experimental conditions, inherent biological complexity, and stochastic molecular interactions can precipitate false positive or negative results, underscoring the imperative for rigorous validation and complementary investigative approaches.

#### 4.2.2. High-Content Screening (HCS) Technology

HCS technology represents a natural evolution from HTS, distinguished by its focus on multiparametric monitoring of cellular activities. It enables the simultaneous detection of target activation, receptor internalization, signal transduction, and a myriad of other biological processes, thereby offering a more nuanced phenotypic readout than traditional HTS [183]. Consequently, HCS is considered a highly promising technique for drug screening. This technology employs advanced high-content imaging systems to capture various aspects of bacterial cells, including morphology, growth dynamics, and metabolic states, following drug treatment. Subsequently, quantitative analysis of this rich information is conducted using sophisticated image analysis software [184]. For example, researchers successfully screened bimatoprost—a prostaglandin F2α analog—from a library of 1017 compounds using HCS imaging. This compound demonstrated potential in ameliorating vancomycin-induced nephrotoxicity and, more importantly, in enhancing the therapeutic efficacy of antibiotics against multidrug-resistant Gram-negative bacteria [185].

#### 4.2.3. CombiANT

CombiANT is an innovative drug screening method based on antibiotic diffusion, which ingeniously creates a unique microenvironment by incorporating three different pairs of antibiotics onto a single agar plate to evaluate synergistic effects [186,187]. This versatile platform can also be applied to investigate the synergy between active compounds and antibiotics. Bland–Altman analysis revealed a minimal deviation of 0.049 between FICI data obtained from the checkerboard method and the CombiANT assay, which closely approaches the detection limit for FICI differences, indicating its high accuracy and comparability. Recent studies have demonstrated CombiANT’s utility in drug screening by exploring interactions between two commonly used aminoglycoside drugs (gentamicin and tobramycin) and two β-lactam drugs (ampicillin and cefotaxime). Surprisingly, among 254 tested strains, none of these four combinations exhibited synergistic effects. Conversely, some strains displayed antagonism when exposed to these combinations, suggesting potential risks associated with empiric combination therapy for bloodstream infections and highlighting CombiANT’s capacity to reveal both synergistic and antagonistic interactions.

#### 4.2.4. Computer-Aided Drug Design (CADD)

Drug virtual screening technology, widely known as CADD, is a sophisticated computational methodology that utilizes simulations to predict drug activity by modeling the interaction process between potential drug candidates and biological targets [188,189]. This cutting-edge technology primarily relies on molecular docking principles to assess the binding affinity and potential pharmacological effects of compounds with their respective targets. Quantitative Structure-Activity Relationship (QSAR) represents one of the most widely employed techniques in virtual screening for predicting antibacterial activity [190]. By establishing robust QSAR models, it becomes possible to accurately forecast the activity levels of unexplored compounds, thereby facilitating the rapid identification and elimination of inactive compounds during the early stages of drug discovery, ultimately saving valuable time and resources [191]. In recent studies, Zhang et al. [115] successfully applied well-established QSAR models to design and synthesize a novel pleuromutilin compound featuring a thiol functional group side chain. The synthesized compound exhibited remarkable antibacterial efficacy against Staphylococcus aureus ATCC 29213 and MRSA, with MIC values below 0.0625 μg/mL. Additionally, Hodyna et al. [192], leveraging various machine learning methods through the OCHEM network platform, developed QSAR models for assessing antibacterial activity in quinoline derivatives. Subsequently, researchers selected and synthesized 2(4)-hydrazone derivatives of quinoline, which exhibited potent antibacterial activity against standard and antibiotic-resistant *S. aureus* and *E. coli* strains, producing growth inhibition zones of 15–30 mm in diameter.

#### 4.2.5. Consensus Virtual Screening Methods

The new generation of drug screening technologies is characterized by its integration of high-throughput capabilities with virtual methodologies. Serafim et al. [193] proposed a consensus virtual screening method that combined holographic quantitative structure-activity relationship (HQSAR) models and molecular docking with computer simulation techniques. Molecular dynamics (MD) simulations were subsequently conducted to screen an impressive 7.6 million compounds, resulting in the identification of 2775 potential hits. From these, five compounds exhibited inhibitory effects ranging from 28 ± 7 to 70 ± 7 μM against test strains. Smiejkowska et al. [194] described the development and validation of a luminescence-based, target-oriented detection method for identifying novel compounds that inhibit Mycobacterium tuberculosis thioredoxin reductase (MtrMtb). This method was utilized for developing highly sensitive and robust HTS assays. Approximately 130,000 compounds were screened using this semi-automated approach, from which 19 were retained after evaluating their potency, selectivity, and specificity. In summary, virtual screening technologies, particularly consensus approaches, offer extensive applications in areas such as new drug development and lead compound selection. They provide powerful tools for rational drug design and discovery, holding significant promise for playing a constructive role in antibiotic adjuvant design and discovery.

#### 4.2.6. Single-Cell Microfluidic Technology

The rapid progress in microfluidic technology is fundamentally driving advancements in drug discovery. The application of single-cell microfluidic technology in drug screening relies on its unique ability to precisely isolate, manipulate, and analyze individual cells [195]. This technology utilizes intricate microchannels and microvalves within microfluidic chips to precisely control cell suspensions, enabling the isolation, cultivation, and detailed analysis of single bacterial cells [196]. Screening drugs at the single-cell level offers a critical advantage by closely mimicking the physiological state of bacteria in situ and minimizing the confounding influence of cellular heterogeneity on experimental results. Moreover, single-cell microfluidic technology often integrates various advanced detection methods, such as fluorescence imaging and electrochemical detection, allowing for real-time monitoring of drug-cell interactions and the acquisition of more comprehensive experimental data. For instance, Shinde et al. [197] successfully observed real-time protein synthesis by detecting changes in fluorescent regions within individual cells. This capability provides researchers with a deeper understanding of drug mechanisms and facilitates the optimization of drug formulations to enhance efficiency and quality during drug development. In a recent study, single-cell microfluidic technology was employed to evaluate berberine hydrochloride (BBH) as an adjuvant for carbapenem antibiotics against multidrug-resistant Acinetobacter baumannii infections. Compared with traditional screening methods, single-cell microfluidic technology provides a more intuitive observation of bacterial morphological changes and dynamic inhibition processes under drug influence, while also facilitating the retrieval of specific cells from the instrument after drug exposure for further in-depth research [198].

## 5. Charting the Future of Antibiotic Adjuvants

Antibiotic resistance poses an existential threat to global public health security, underscoring the urgent imperative for innovative strategies to combat drug-resistant bacterial infections. This review has systematically elucidated the diverse classifications, intricate mechanisms of action, and evolving screening methodologies for antibiotic adjuvants, revealing their immense potential in restoring the efficacy of existing antibiotics and directly confronting multidrug-resistant bacteria. The rich tapestry of potential adjuvant sources spans natural products, exquisitely designed synthetic molecules, targeted bacteriophages, and repurposed clinical drugs. This breadth offers a robust foundation for identifying compounds that can strategically disarm bacterial resistance mechanisms.

To summarize the diverse landscape of these agents, Table 2 outlines the primary advantages and critical limitations inherent to each major category of antibiotic adjuvants. The successful translation of these strategies is already becoming a reality, as evidenced by recent clinical milestones. For example, the novel β-lactam/β-lactamase inhibitor combination sulbactam-durlobactam was approved by the US FDA in May 2023 specifically to target carbapenem-resistant Acinetobacter baumannii (CRAB). Similarly, cefepime-enmetazobactam was approved in early 2024 for complicated urinary tract infections, demonstrating clinical superiority against resistant pathogens. Concurrently, the integration of Artificial Intelligence (AI) is rapidly accelerating the discovery of next-generation adjuvants. Deep learning models and AI-driven platforms are now being utilized to mine vast chemical spaces and predict novel synergistic pairs, drastically reducing early-stage discovery time and costs.

Their mechanisms of action are remarkably diverse and sophisticated, encompassing the inhibition of resistance enzymes, suppression of efflux pump activity, modulation of bacterial membrane permeability, metabolic reprogramming, interference with critical signal transduction pathways, and disruption of protective biofilms. This multi-target, multi-pathway approach reflects the nuanced and precise strategies adjuvants employ to reverse bacterial resistance at multiple levels, ultimately enhancing antibiotic susceptibility. Furthermore, the synergistic integration of advanced technologies, such as high-throughput screening and computer-aided drug design, with traditional screening methods provides unprecedented technical support and opens novel avenues for the efficient discovery and optimization of these critical compounds.

Despite this compelling promise, the clinical translation of antibiotic adjuvants faces several formidable challenges. The mechanistic intricacies of some adjuvants remain incompletely elucidated, necessitating deeper molecular-level understanding. Comprehensive studies are still required to fully characterize their safety profiles, in vivo stability, and pharmacokinetic/pharmacodynamic properties. Moreover, determining the optimal dosage ratios, administration routes, and precise applicable strain ranges for adjuvant-antibiotic combinations demands rigorous validation through extensive preclinical and clinical trials. Crucially, the long-term risk of inducing novel bacterial resistance to adjuvants themselves requires continuous monitoring and the proactive development of adaptive response strategies.

Addressing these challenges necessitates a focused, multi-pronged research agenda. Firstly, future efforts must concentrate on elucidating the molecular underpinnings of adjuvant action, providing a robust theoretical framework for their precise design and rational optimization. Secondly, the development of more efficient and highly specific screening platforms is essential to accelerate the discovery pipeline for novel adjuvants. Thirdly, rigorous preclinical and clinical validation of adjuvant-antibiotic combinations is paramount to establish their efficacy, safety, and clinical utility across diverse infectious disease settings. Finally, the implementation of robust, real-time drug resistance surveillance networks is critical to track changes in bacterial susceptibility during adjuvant application, allowing for timely intervention and adaptation.

In summary, antibiotic adjuvants offer a practical and highly promising approach to navigate the complexities of bacterial resistance. However, their successful transition from laboratory bench to widespread clinical practice will demand concerted interdisciplinary collaboration and sustained scientific exploration. By continuously refining the theoretical foundations, optimizing technical methodologies, and strengthening clinical validation, antibiotic adjuvants are poised to play a transformative role in future anti-infective treatments, making an indispensable contribution to mitigating the global crisis of antimicrobial resistance.

## Figures and Tables

**Table 1:** The types and mechanisms of action of antibiotic adjuvants.

Category	Natural Products			Artificially Synthesized Molecules		Bacteriophages	Clinical Therapeutic Drugs
Type	*Phytochemicals*	*Animal-Derived Compounds*	*Microbial-Derived Compounds*	*Nanomaterials*	*Synthetic Peptides*	*Bacteriophage-Antimicrobial Combinations*	*Repurposed Drugs*
Examples	Berberine, Matrine, Flavonoids	L-lysine, Melatonin, Antimicrobial peptides	Glucose, Mannitol, Fructose	Silver nanoparticles, Chitosan	dUSTBP8, Nisin	vB_3530, vB_1086	N-acetylcysteine, Metformin, Oxyclozanide
Mechanism of Action	Biofilm disruption, membrane permeabilization, antibiotic synergy	Enhance outer membrane permeability, oxidative stress induction	Metabolic reprogramming, enhancement of antibiotic uptake	Disruption of bacterial membranes, inhibition of cell-to-cell communication	Synergistic effects with antibiotics, altering bacterial resistance mechanisms	Potent antibacterial activity, inhibition of biofilm formation	Disruption of bacterial membranes, enhancing antibiotic uptake
Applications	Enhancing efficacy of β-lactams, tetracyclines, and others	Improving susceptibility of multidrug-resistant pathogens	Potentiating effectiveness against resistant strains	Reducing biofilm formation, enhancing antibiotic activity	Targeting Gram-positive and Gram-negative bacteria	Addressing antibiotic resistance through phage therapy	Overcoming resistance in various bacterial strains

**Table 2:** Advantages and Limitations of Key Antibiotic Adjuvant Strategies.

Strategy Category	Key Advantages	Primary Limitations
**Natural Products**	High structural diversity; generally low mammalian toxicity; often possess multi-target mechanisms (e.g., biofilm disruption and ROS induction).	Poor or variable bioavailability; complex extraction and purification processes; challenges in standardization.
**Synthetic Molecules**	Precise structural design and optimization; high targeted efficacy; easily scalable and reproducible production.	Potential for off-target human toxicity (e.g., cell membrane damage); high initial research and synthesis costs.
**Bacteriophages**	Extreme target specificity (preserves commensal microbiome); self-replicating at the infection site; highly effective against biofilms.	Rapid clearance by the host immune system; narrow host range necessitates personalized cocktails; complex regulatory hurdles.
**Repurposed Drugs**	Established safety profiles and known pharmacokinetics in humans; significantly accelerated clinical approval pathways.	Required efficacy against bacteria often demands high, off-label dosages; potential for unintended off-target systemic side effects.

## Data Availability

No new data were created or analyzed in this study. Data sharing is not applicable to this article.

## Abbreviations

The following abbreviations are used in this manuscript:

AMR	antibiotic resistance
MDR	multidrug-resistant
VRE	vancomycin-resistant *Enterococci*
MRSA	methicillin-resistant *Staphylococcus aureus*
CRE	carbapenem-resistant *Enterobacteriaceae*
PDR	pan-drug-resistant
BER	Berberine
MIC	minimum inhibitory concentration
ROS	reactive oxygen species
EGCG	epigallocatechin gallate
EOs	Essential oils
PMF	proton motive force
TCSs	two-component signal transduction systems
FICI	fractional inhibitory concentration indices
HTS	high-throughput screening
HCS	High-Content Screening
